# Effects of selenium, selenium‐enriched spirulina and phycocyanin on myocarditis stress parameters on LPS‐induced injury in H9c2 ventricular cardiomyoblasts

**DOI:** 10.14814/phy2.70376

**Published:** 2025-05-19

**Authors:** Thomas Castel, Karine Pichavant‐Rafini, Michaël Théron, Charlotte Gandubert, Karelle Léon

**Affiliations:** ^1^ Univ Brest, Laboratory ORPHY, IBSAM Brest France; ^2^ LBAI, UMR1227 Univ Brest, Inserm Brest France; ^3^ CHU de Brest Brest France

**Keywords:** cardiac inflammation, phycocyanin, selenium, spirulina

## Abstract

Myocarditis is strongly represented in septic patients and is associated with a higher mortality rate. *Spirulina platensis* (Spi), a blue‐green algae, has anti‐inflammatory properties and can be enriched with selenium, an antioxidant essential oligoelement. In addition, phycocyanin (PC), a biliprotein extract from spirulina, displays interesting anti‐inflammatory and antiapoptotic effects. In this study, the objective was to determine the cardioprotective effects of Sodium selenite (Se), Spi, Spi + Se (SeSP) and PC on LPS‐induced inflammation, apoptosis, and oxidative stress parameters. H9c2 cells were co‐treated with or without LPS (5 μg/mL) and Se (0.5 μM), Spi (2.5 μg/mL), SeSP (0.5 μM Se + 2.5 μg/mL Spi) and PC (0.1 μg/mL) for 24 h. Inflammation was investigated by measurement of NFκB activation, IL‐6, and caspase 1 expression, while apoptosis was measured by Bax, Bcl‐2, and caspase‐3 expression. Furthermore, GPx and SOD activities were analyzed, as well as isoprostanes and nrf‐2 expression. Activation of MAPK Junk and p38 was also determined. Our results demonstrated that Se could only reduce p65 S536 phosphorylation. SeSP could limit Bax expression, while an increase in IL‐6 was detected without LPS. Moreover, PC could reduce IL‐6 and Caspase‐1 expression and could have promising properties to decrease LPS‐induced myocarditis outcomes.

## 1 INTRODUCTION

In 2016, during the Third International Consensus of Sepsis Definition, sepsis was defined as life‐threatening organ dysfunction caused by a dysregulated host response to infection (Singer et al., 2016). It is the leading cause of mortality in intensive care units and was responsible for 11 million deaths in 2017 (Rudd et al., 2020). In sepsis, immune cells recognize specific infectious agents, such as LPS of bacterial wall, through their TLR4 receptors (Kumar, 2020). This recognition leads to an inflammatory and an oxidative stress response which, although essential to fight the infection, will strongly affect the host. Thus, LPS addition is one of the most common models of sepsis induction in vivo and is used as an inflammation stimulation in vitro. The cardiovascular system is highly impacted during sepsis (Antonucci et al., 2014). A disruption of cardiomyocyte (a cell type exhibiting TLR4 receptors) metabolism as well as damage related to inflammation and oxidative stress could be at the origin of a cardiomyopathy (L'Heureux et al., 2020). This cardiomyopathy results in ventricular impairment, insufficient cardiac output, and oxygen delivery (Beesley et al., 2018). Finally, septic shock, the most advanced stage of sepsis, is associated with the necessity of using vasopressors to maintain a mean arterial pressure above 65 mmHg. Numerous studies have focused on cardiac failure during sepsis, but its pathophysiology remains poorly understood.

Selenium is an essential trace element involved in antioxidant defenses due to its incorporation in selenoproteins such as glutathione peroxidase. It has been shown that 92% of septic patients are selenium deficient (Sakr et al., 2007) and that selenium concentration appears to decrease during the sepsis course (Renko et al., 2009). Moreover, selenium deficiency and ROS are also involved in cardiomyopathy development. Indeed, ROS can lead to contractile dysfunction, ventricular arrhythmias, and myocardial impairments (Neri et al., 2016). Study selenium is hence particularly interesting in the case of sepsis.

Spirulina is a blue‐green algae known for its nutritional properties. Rich in proteins, vitamins, and pigments, spirulina is used as a dietary supplement. However, an increasing number of studies aim to demonstrate its therapeutic effects. Indeed, spirulina has anti‐inflammatory activity mainly via reduction of cytokine release such as IL‐6, TNF‐α or NF‐κB pathway inhibition (Morsy et al., 2019; Zahran & Emam, 2018). Furthermore, spirulina can also act as an antioxidant. An increase in antioxidant enzyme activities and a decrease in oxidative stress markers are found in many animal models after spirulina intake (Karadeniz et al., 2008; Mahmoud & Abd El‐Ghffar, 2019). Moreover, spirulina can be enriched in selenium during its culture.

Phycocyanin (PC) is a major photosynthetic pigment present in spirulina. In recent years, studies have shown that PC has many therapeutic properties such as anti‐inflammatory (Alzokaky et al., 2020), antioxidant (Romay et al., 2003), antiapoptotic (Kim et al., 2018) and anti‐cancer (Jiang et al., 2017; Kaur et al., 2020) in various animal and in vitro models. Furthermore, PC can prevent cardiac impairments in an ischemia/reperfusion model by acting on Mitogen‐activated protein kinases (MAPK) activities (Khan et al., 2006).

Inflammation and oxidative stress persistence are directly associated with the worsening of septic patients' condition. Thus, reducing these aspects and discovering new therapeutic approaches are a priority. The concomitant effects of spirulina and selenium on inflammation and oxidative stress could reduce these bad outcomes. Moreover, phycocyanin, present in spirulina, also seems to be promising in the regulation of inflammation, oxidative stress, and apoptosis. In this context, the objective of this study was to evaluate the potential beneficial effect of selenium, spirulina extract, selenium associated with a spirulina extract, or phycocyanin on inflammation, oxidative stress, and apoptosis parameters of cardiac cells (H9c2) under stress conditions (with LPS). Furthermore, we investigated the effects of these supplementations on cellular viability in H9c2 cells.

## 2 MATERIALS AND METHODS

### 2.1 Reagents

LPS and sodium selenite (Na_2_SeO_3_) were provided by Sigma‐Aldrich (St. Louis, MO, USA). The spirulina strain used in this study was *Spirulina platensis*. Production and conditioning (as dried powder) of spirulina were carried out by TAM company (Plougastel, France). Spirulina powder was dissolved in water and sonicated for 30 min. Then, spirulina was frozen, thawed 3 times, and centrifuged for 5 min at 3400 ***g***. Then, another cycle of freeze–thaw was performed, and spirulina was centrifuged for 5 min at 8000 ***g***. Spirulina was finally diluted at 2.5 μg/mL in DMEM with 1% fetal bovine serum. Phycocyanin was provided by Algaenutri Company. Phycocyanin extract was diluted to 0.1 μg/mL in DMEM 1%.

### 2.2 Assessment of cellular viability

**FIGURE 1:**
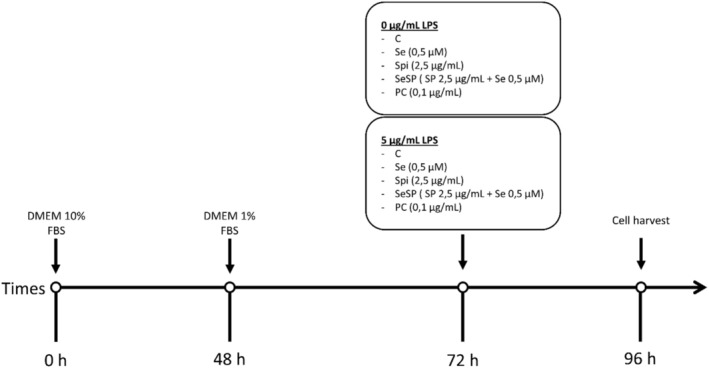
Experimental protocol.

Cells were seeded at 1 × 10^6^ cells/mL on 96‐well culture plates and received the same treatment as described above (Figure 1). At 72 h, LPS (0, 5, 10, 15, 45, 60 or 240 μg/mL), Selenium as sodium selenite (0, 1, 2.5, 5, 10, 20, 40, 60 or 80 μM), spirulina extract (0, 1, 2.5, 5, 7.5, 10, 12.5, or 15 μg/mL) or phycocyanin (0, 0.1, 1, 2.5, 5, 7.5, 10, 12.5 or 15 μg/mL) were added and incubated at 37°C for 24 h. Then, culture medium was replaced by 100 μL of MTT solution (5 mg/mL) for 2 h and incubated at 37°C. Finally, absorbance was measured at 450 and 540 nm using a microplate reader. All results are expressed as percent of control (without interest compounds). For this study, 5 μg/mL LPS, 0.5 μM Se, 2.5 μg/mL Spi, and 0.1 μg/mL PC have been chosen.

### 2.3 Cell culture and treatment

The rat H9c2 cell line, derived from embryonic rat heart ventricle, was purchased in ATCC cell line. Cells were cultivated in Dulbecco's modified Eagle's medium (DMEM) (Biowest, L0093‐500) supplemented with 10% fetal bovine serum, 100 UI/mL penicillin, and 100 μg/mL streptomycin (DE17‐602E, Lonza). Cells were incubated in a humidified atmosphere containing 95% air and 5% CO_2_ at 37°C.

Cells were cultured for 24 h with DMEM 1% fetal bovine serum in order to synchronize the cells. Then, cultured cells were divided into different groups: Control (C), selenium as sodium selenite 0.5 μM (Se), spirulina extract 2.5 μg/mL (Spi), selenium as sodium selenite 0.5 μM + spirulina extract 2.5 μg/mL (SeSP) and Phycocyanin 0.1 μg/mL (PC). All groups were divided into two subgroups, with or without LPS 5 μg/mL. LPS was added at the same time as supplementations, and each supplementation lasted for 24 h (Figure 1).

### 2.4 Inflammation, apoptosis and oxidative stress parameters analysis

Supernatant IL‐6 levels were measured by ELISA according to the manufacturer's instructions (R&D biosystems, R600B, pg/mL). Phosphorylation of p65 Serine 536 (PEL‐NF‐κBP64‐S536, Raybiotech, Optical Density) and p65 total were determined by an ELISA kit (E‐EL‐R0674, Elabscience, pg/mL). Caspase 1 levels were determined by an ELISA test (MBS7700529, MyBioSource, U/mL).

Bax (ng/mL), Bcl‐2 (ng/mL) and caspase 3 (ng/mL) levels were measured by ELISA test according to the manufacturer's instructions (E‐EL‐R0098; E‐EL‐R0096; E‐EL‐R0160, Elabscience).

GPx activity was measured at 340 nm with an indirect method adapted from Ross et al. (2001) by Farhat et al. (2015). Briefly, the activity was determined from the decrease of NADPH induced by a coupled reaction with glutathione reductase. GPx activity was expressed in nmol NADPH oxidized/min/mg of proteins (U/mg prot).

SOD activity was measured using the Total SuperOxide Dismutase (T‐SOD) activity Assay Kit (WST‐1 Method) according to the manufacturer's instructions (E‐BC‐K020‐M) (U/mg prot).

Nrf2 (pg/mL) and isoprostanes (pg/mL) levels were measured by ELISA kit (E‐EL‐R1052; E‐EL‐0041, Elabscience).

Junk and p38 levels were measured by ELISA test according to manufacturer's instructions (ab207477; ab207483, Abcam, Arbitrary Unit).

### 2.5 Statistical analysis

All results are expressed as mean ± standard error of mean (SEM). Normality was tested using the Shapiro–Wilk test. For the cellular viability analysis experiment, ANOVA test was performed. For inflammatory, antioxidative, and apoptotic parameters, adapted tests were performed (Student or Mann–Whitney tests). A *p* value <0.05 was considered significantly different. Moreover, principal component analysis was used to investigate the correlation between the parameters following LPS addition. Statistics and PCA were performed with GraphPad Prism v9.0.2 software.

## 3 RESULTS

### 3.1 Cellular viability analysis

**FIGURE 2:**
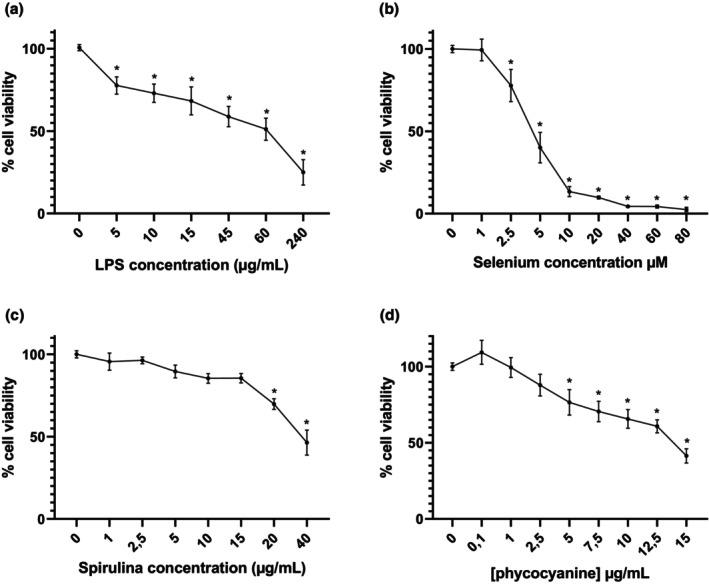
Effects of LPS (a), selenium (b), spirulina extract (c), and phycocyanin (d) on H9c2 cells viability. Data are expressed as mean ± SEM (*n* = 6 for each condition). Anova test was performed, and * indicates significant difference with 0 of each supplementation (*p* < 0.05).

The first step was to determine the working concentration of each of our supplements by the realization of dose–response survival curves. Viability analysis at different concentrations in different conditions is presented in Figure 2. A reduction in cell viability is observed from 25% at 5 μg/mL to 50% at 60 μg/mL LPS and 75% at 240 μg/mL LPS (Figure 2a). For sodium selenite, no significant difference in cell viability was observed at 1 μM. However, a 25% reduction in cell viability was observed at 2.5 μM sodium selenite (Figure 2b). No change in viability was observed for concentrations below 20 μg/mL for spirulina extract. At 20 μg/mL, a reduction of 30% and 55% at 40 μg/mL of spirulina can be noted (Figure 2c). Finally, phycocyanin has no effect on viability up to 2.5 μg/mL while a 25% reduction is observed at 5 μg/mL (Figure 2d). Thus, in this study, a concentration of 5 μg/mL of LPS was chosen. Regarding the experimental conditions, our study was conducted at 0.5 μM Se, 2.5 μg/mL Spi, and 0.1 μg/mL PC.

### 3.2 Inflammation parameters measurements

**FIGURE 3:**
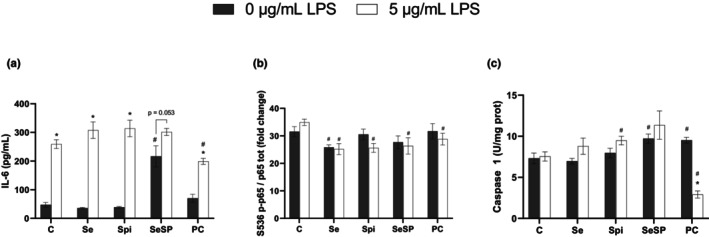
Effects of supplementations on inflammation parameters after LPS addition. IL‐6 (a), S536 p65/p65 (b) and Caspase 1 (c) were measured with or without 5 μg/mL of LPS and different types of supplementations (Se, Spi, SeSP or PC). Data are expressed as mean ± SEM (*n* = 6 for each condition). C, Control; Se, Selenium 0.5 μM; Spi, Spirulina extract 2.5 μg/mL; SeSP, Selenium 0.5 μM + spirulina extract 2.5 μg/mL; PC, Phycocyanin 0.1 μg/mL. Student test were performed for IL‐6 and S536 p‐p65/S536 p65, and Mann–Whitney test was performed for Caspase 1. * indicates a significant difference with 0 μg/mL of LPS in the same group (*p* < 0.05) and # indicates a significant difference with the Control condition at the same concentration of LPS.

Without LPS, no significant difference in IL‐6 concentration was observed between the control condition and Se, Spi, or PC (Figure 3a). However, an increase in IL‐6 concentration was observed when the cells were exposed together to the spirulina extract (2.5 μg/mL) and the sodium selenite (0.5 μM) with a level of 216.5 ± 37.0 pg/mL compared to control 47.5 ± 8.1 pg/mL. At 5 μg/mL LPS, a significant increase in IL‐6 concentration was observed in all conditions compared to the control condition without LPS, except for SeSP where the IL6 increase with LPS was not significant (*p* = 0.053). In the presence of LPS, it is interesting to note that PC showed a significantly lower IL‐6 concentration (198.9 ± 10.7 pg/mL) than the control (259.2 ± 15.5 pg/mL).

Regarding the phosphorylation of p65 S536, only Se showed a decrease at 0 μg/mL LPS compared to the control without LPS (Figure 3b). At 5 μg/mL LPS, all experimental conditions displayed a decrease in p65 S536 phosphorylation compared to the control.

At 0 μg/mL LPS, caspase 1 levels (Figure 3c) showed a significant increase in the presence of SeSP (9.7 ± 0.6 U/mg prot) and PC (9.5 ± 0.4 U/mg prot) compared to the control (7.3 ± 0.6 U/mg prot). On the other hand, in the presence of 5 μg/mL LPS, only Spi (9.5 ± 0.5 U/mg prot) showed an increase compared to the control (7.5 ± 0.6 mg prot) while caspase 1 levels in the presence of PC (2.9 ± 0.4 U/mg prot) were drastically reduced.

### 3.3 Apoptosis parameters analysis

**FIGURE 4:**
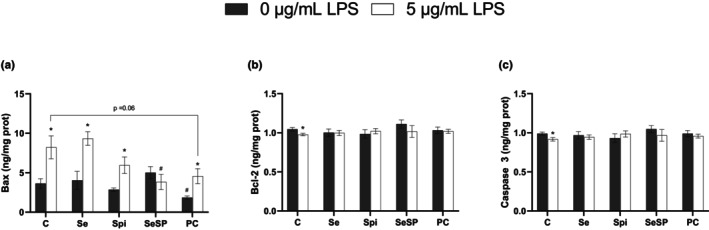
Effects of supplementations on apoptosis parameters after LPS addition. Bax (a), Bcl‐2 (b) and Caspase 3 (c) were measured with or without 5 μg/mL of LPS and different types of supplementations (Se, Spi, SeSP or PC). Data are expressed as mean ± SEM (*n* = 6 for each condition). C, Control; Se, Selenium 0.5 μM; Spi, Spirulina extract 2.5 μg/mL; SeSP, Selenium 0.5 μM + spirulina extract 2.5 μg/mL; PC, Phycocyanin 0.1 μg/mL. Student test was performed for all the parameters. * indicates a significant difference with 0 μg/mL of LPS in the same group (*p* < 0.05) and # indicates a significant difference with the Control condition at the same concentration of LPS (*p* < 0.05).

At 0 μg/mL LPS, Bax concentration displayed no significant difference between the experimental conditions and the control (3.6 ± 0.6 ng/mg prot) except for PC (1.8 ± 0.2 ng/mg prot) which showed reduced Bax (Figure 4a). On the other hand, an increase in the concentration of Bax was observed at 5 μg/mL of LPS for the control, Se, Spi, and PC conditions compared to their own levels without LPS. In the presence of LPS, Bax level was significantly reduced by SeSP incubation (from 8.2 ± 1.4 in the control group to 3.8 ± 0.9 ng/mg prot in SeSP). In the same condition, the effect of PC incubation was similar but did not reach statistical significance (*p* = 0.06, 4.5 ± 0.9 ng/mg prot).

Concerning Bcl‐2 levels (Figure 4b), no significant difference appeared between experimental conditions compared to control (1.0 ± 0.02 ng/mg prot) at 0 μg/mL LPS. The only effect observed was, in the control condition, a reduction of bcl‐2 (0.97 ± 0.01 ng/mg prot) following the addition of 5 μg/mL of LPS.

Caspase 3 levels (Figure 4c) displayed the same profile as Bcl‐2. Indeed, no significant difference at 0 μg/mL LPS in all experimental conditions was shown, but a slight reduction in the control group (0.91 ± 0.02 ng/mg prot) at 5 μg/mL LPS compared to 0 μg/mL LPS (0.98 ± 0.02 ng/mg prot) was observed.

### 3.4 Oxidative stress parameters analysis

**FIGURE 5:**
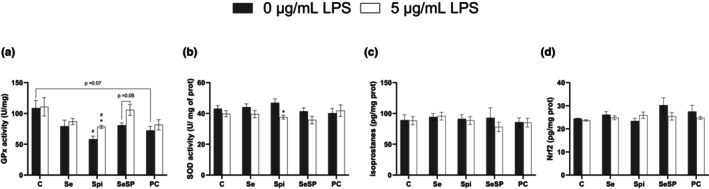
Effects of supplementations on oxidative stress parameters after LPS addition. GPx activity (a), SOD activity (b) isoprostanes (c) and Nrf‐2 (d) were measured with or without 5 μg/mL of LPS and different types of supplementations (Se, Spi, SeSP or PC). Data are expressed as mean ± SEM (*n* = 6 for each condition). C, ControlL; Se, Selenium 0.5 μM; Spi, Spirulina extract 2.5 μg/mL; SeSP, Selenium 0.5 μM + spirulina extract 2.5 μg/mL; PC, Phycocyanin 0.1 μg/mL. Mann–Whitney tests were performed for GPx and Nrf2, and Student tests were performed for SOD activity and isoprostanes. * indicates a significant difference with 0 μg/mL of LPS in the same group (*p* < 0.05) and # indicates a significant difference with the Control condition at the same concentration of LPS (*p* < 0.05).

Oxidative stress parameters analysis is presented in Figure 5. Without LPS, GPx activity (Figure 5a) is reduced in the Spi group (58.1 ± 5.2 U/mg prot) compared to the control group (108.5 ± 12.6 U/mg prot). In the same condition (no LPS), the other supplementations did not induce any significant difference with the control group. Nevertheless, GPx activity was slightly reduced with phycocyanin (72.1 ± 6.7 U/mg prot, *p* = 0.07) compared to the 0 μg/mL LPS control. Only Spi displayed a rise in GPx activity (78.2 ± 2.4 U/mg prot) following LPS addition, while SeSP showed an increasing non‐significant trend (*p* = 0.06). At 5 μg/mL LPS, GPx activity was reduced in Spi (78.3 ± 2.4 U/mg prot) compared to the control group (110.7 ± 14.9 U/mg prot).

There was no significant difference between groups neither with nor without LPS concerning SOD activity (Figure 5b). The only difference observed was a reduction in SOD activity following LPS addition in Spi group.

Concerning isoprostanes levels, no significant difference appeared between groups at 0 and 5 μg/mL LPS (Figure 5c). Nrf2 levels displayed the same profile with no significant difference in any groups and LPS concentrations (Figure 5d).

### 3.5 MAPK quantification analysis

**FIGURE 6:**
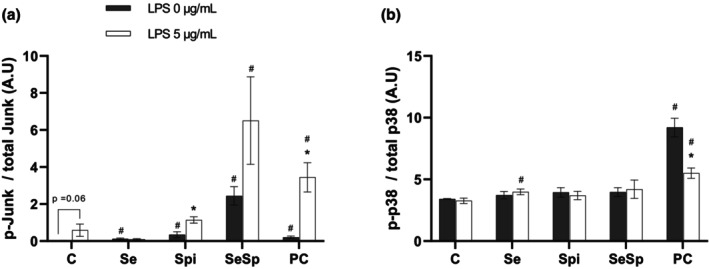
Effects of supplementations on MAPk pathway activation after LPS addition. Junk activation (p‐Junk/total Junk ratio) (a) and p38 activation (p‐p38/total p38 ratio) (b) were measured with or without 5 μg/mL of LPS and different types of supplementations (Se, Spi, SeSP or PC). Data are expressed as mean ± SEM (*n* = 6 for each condition). C, Control; Se, Selenium 0.5 μM; Spi, Spirulina extract 2.5 μg/mL; SeSP, Selenium 0.5 μM + spirulina extract 2.5 μg/mL; PC, Phycocyanin 0.1 μg/mL. Mann–Whitney test was performed for JNK and Student's test was performed for p38. * indicates a significant difference with 0 μg/mL of LPS in the same group (*p* < 0.05) and # indicates a significant difference with the Control condition at the same concentration of LPS (*p* < 0.05).

Activation of JUNK and p38 pathway are represented in Figure 6. Concerning JUNK activation (Figure 6a), Se (0.12 ± 0.03 AU), Spi (0.35 ± 0.15 AU), SeSP (2.44 ± 0.50 AU) and PC (0.21 ± 0.06 AU) induced an increase in JNK activation at 0 μg/mL LPS compared to the control (0.03 ± 0.01 AU). Spi (1.14 ± 0.17 AU) and PC (3.44 ± 0.78 AU) JNK activation is increased following LPS addition, while Se and SeSP displayed no significant difference. Moreover, the control group showed a non‐significant trend to increase at 5 μg/mL LPS (*p* = 0.06). At 5 μg/mL LPS, only SeSP and PC showed an increased JNK activation compared to the control group (0.59 ± 0.33 AU).

At 0 μg/mL, only PC displayed a significant increase in the p‐p38/total p38 ratio (9.20 ± 0.75 AU) compared to the control group (3.41 ± 0.04 AU) (Figure 6b). Following exposure to 5 μg/mL LPS, PC appeared to largely reduce p38 activation (5.50 ± 0.42 AU) while remaining higher than control (3.26 ± 0.22 AU).

### 3.6 Principal component analysis

**FIGURE 7:**
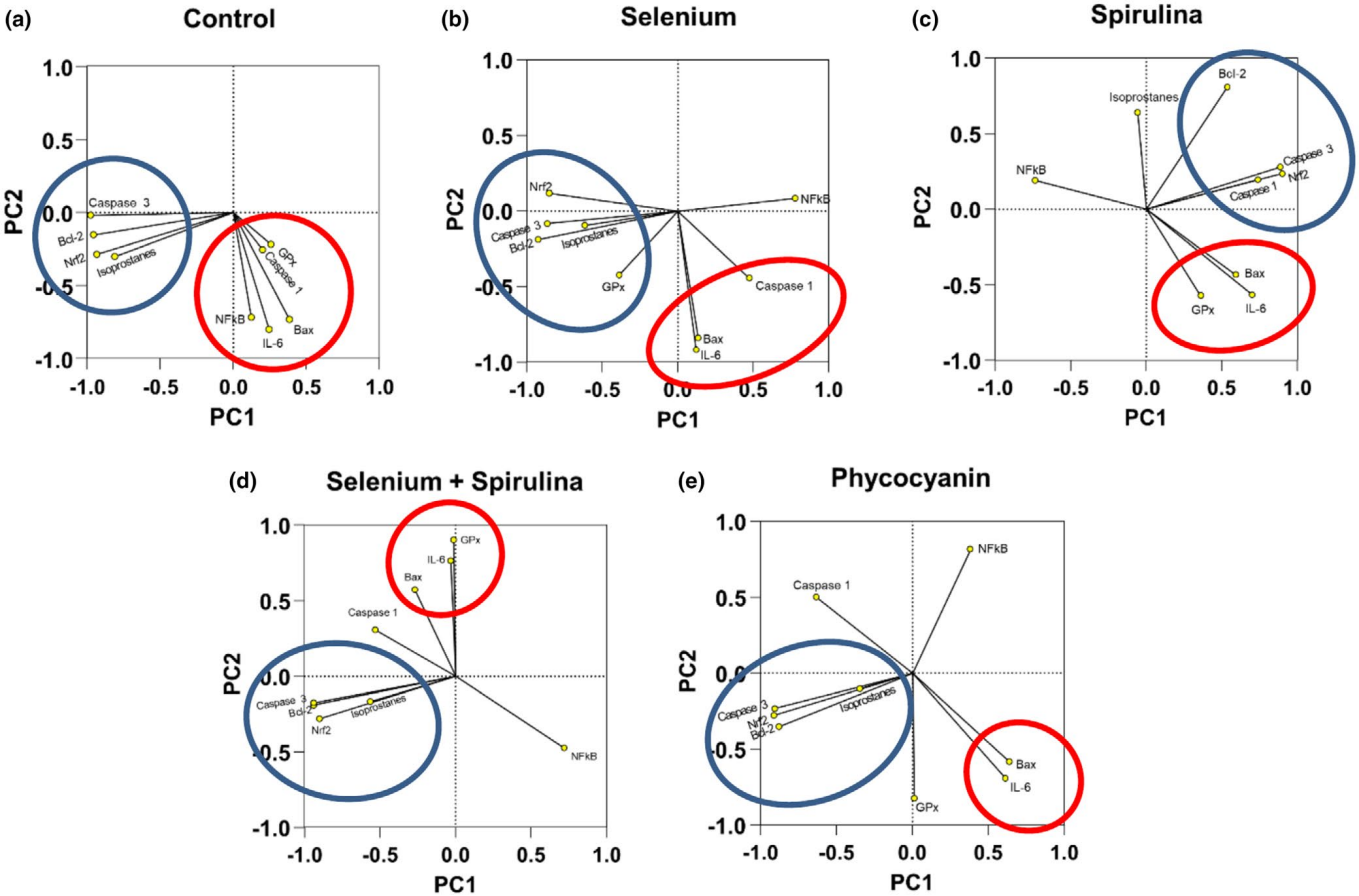
Principal Component Analysis (PCA) of 9 variables following LPS addition in Control (a), Selenium (b), Spirulina (c), Selenium + spirulina (d) and Phycocyanin (e) conditions (*n* = 6 for each condition).

Results of principal component analysis were shown on Figure 7. In the control condition, two orthogonal groups of parameters appeared. The first one was composed of Caspase 3, Bcl‐2, Nrf2, and isoprostanes, while the second one gathered GPx, Caspase 1, Bax, IL‐6, and NF‐κB. The selenium group displayed the same profile except for NF‐κB. Spirulina also showed 2 groups of parameters, but isoprostanes and NF‐κB were not included in these groups anymore. Concerning SeSP, caspase 1 and NF‐κB no longer belonged to one of these two groups. Finally, PC demonstrated 3 parameters outside of these groups: GPx, Caspase 1, and NF‐κB.

## 4 DISCUSSION

Sepsis occurs as a result of infection and is associated with major organ dysfunctions, particularly of the cardiovascular system. Cardiomyocyte metabolism and integrity are particularly affected, contributing to this cardiac dysfunction. Furthermore, mitochondrial dysfunctions are also linked to cardiomyocyte functional impairments (Fan et al., 2025; Torres et al., 2025) In this context, the objective of this study was to determine the effects of supplements (Sodium selenite, spirulina, sodium selenite + spirulina and phycocyanin) known for their anti‐inflammatory, antioxidant, and anti‐apoptotic activities on rat ventricular cardiomyoblasts exposed to LPS.

In this study, Se, Spi, SeSP, and PC were added to H9c2 cells at the same time as LPS. As stated earlier, the first step was the realization of dose–response survival curves. LPS induced a significant reduction in the survival rate in H9c2 cells above 5 μg/mL. These results seem to be in accordance with the literature, which nevertheless shows a wide range of responses for LPS concentrations varying from 0.1 to 10 μg/mL. In fact, some studies displayed a 35% viability reduction after 24 h of LPS exposition at 1 μg/mL (Luo et al., 2018) while others showed a 50% cell viability decrease after 6 h at 1 μg/mL LPS exposure (Li et al., 2020). Concerning sodium selenite, a reduction in viability is observed from 2.5 μM. These results are in agreement with those obtained by Sun et al. (2020) who showed no reduction in H9c2 viability after 24 h of sodium selenite incubation up to 2 μM (Sun et al., 2020). However, few studies have been conducted to determine the effects of spirulina and phycocyanin on H9c2 cells. Jadaun et al. (2018) demonstrated that no viability reduction was observed in the presence of 1, 2, 4, and 8 μg/mL of spirulina extract (Jadaun et al., 2018). In 2019, Gao et al., for their part, showed that phycocyanin enabled an improvement in H9c2 viability in the presence of 10, 20, 40, 60, and 80 μg/mL phycocyanin in an OGD/R (oxygen–glucose deprivation/reoxygenation) model (Gao et al., 2019). Nevertheless, to our knowledge, no survival curve with phycocyanin only was realized.

In sepsis, the infection leads to a severe inflammation in the organism. Cells recognize specific infectious agents, such as LPS, through their TLR4 receptors. Once activated, the TLR4 pathway leads to the expression of pro‐inflammatory cytokines such as interleukin 6 via NF‐κB activation. These receptors are found on many cells, including cardiomyocytes. NF‐κB is a dimeric transcription factor present in the cytoplasm and plays a key role in the inflammatory response. The NF‐κB family is composed of 5 monomers, p65 (RelA), RelB, c‐Rel, p50, and p52 (Oeckinghaus & Ghosh, 2009) which can associate in homo‐ or heterodimers. The most abundant form is the p50/p65 dimer (Giridharan & Srinivasan, 2018) which is sequestered in the cytoplasm by the IκB protein. Upon TLR4 activation, the IκB protein is phosphorylated and subsequently degraded by the proteasome. The p50/p65 dimer is the target of numerous post‐translational modifications such as acetylation or phosphorylation. It has been shown in JB6 mouse epithelial cells that a decrease in phosphorylation of S536 of p65 is associated with a reduction in the inflammatory response following TNF‐α stimulation (Hu et al., 2004). Interestingly, our results indicate that exposure to 5 μg/mL LPS in H9c2 cells results in a clear IL‐6 release, but this effect appears to be independent of p65 S536 phosphorylation. Moreover, sodium selenite even seemed to induce a decrease of p65 S536 phosphorylation at 0 μg/mL of LPS, whereas all the supplements induced a decrease at 5 μg/mL of LPS compared to the control group. These results suggest that p65 S536 phosphorylation is not involved in interleukin‐6 expression in H9c2 cells. These results are in agreement with the work of Ghosh et al. (2010) who demonstrated that unphosphorylated p65 is associated with IL‐6 promoters, while the phosphorylated form is not in LPS‐stimulated macrophages (Ghosh et al., 2010). Caspase 1 is a component of the inflammasome complex and is associated with the maturation of IL‐1β and IL‐18. Our results demonstrated that phycocyanin was able to significantly reduce the levels of caspase 1 following LPS exposure compared to the control. Furthermore, exposition of 5 μg/mL LPS displayed no significant difference in control conditions, which suggests that caspase 1 was not involved in the inflammatory response in H9c2 cells.

Concerning apoptosis, our results demonstrated a marked increase in Bax expression following exposure to LPS in all conditions except in SeSP. It has been shown that in C57 mice myocardium, exposure to Coxsackie B3 virus induced an IL‐6 overexpression which is responsible for the Bax/bcl‐2 ratio increase via the IL6‐R/STAT3 pathway (Li et al., 2019). Our results seemed to be in agreement with this finding since the profile of Bax corresponds to the IL‐6 one. Thus, the increase in pro‐apoptotic parameters could result from LPS‐induced IL‐6 rise. However, PC seemed to induce a non‐significant trend to reduce Bax levels following LPS exposure compared to the control condition (*p* = 0.06) while SeSP induced a significant reduction, showing a potential anti‐apoptotic effect not mediated by Bcl‐2 expression. In our study, Caspase 3 expression is reduced following LPS exposition in the control condition (while the other supplementation displayed no significant effect). However, Bcl‐2 expression was not affected by LPS exposure in any condition compared to the control group. Thus, caspase 3 expression does not seem to be impacted by LPS or experimental conditions. Additional parameters should be determined, and flow cytometry analysis could be performed to investigate if the LPS‐induced apoptosis is caspase 3 independent.

Oxidative stress is a cellular process that can occur as a secondary result of inflammation. It is characterized by an imbalance between radical species production and antioxidants. GPx and SOD constitute two main antioxidant enzymes. In our study, our results indicated that LPS addition did not increase GPx activity in any conditions except for SeSP (with a trend toward an increase). This is surprising for the selenium condition since GPx is a selenoprotein. We hypothesized that the selenium concentration in the culture medium was sufficient to maintain basal GPx activity. Moreover, only spirulina seemed to show a tendency to reduce GPx activity following LPS exposure. However, this could be a compensation of the increased SOD activity. Isoprostanes (produced from lipid peroxidation of arachidonic acid) are a good marker of oxidative stress (Cracowski, 2004). In addition, nrf2 is a transcription factor that is responsible for the expression of many antioxidant genes. Under normal conditions, it is sequestered in the cytosol and then degraded by the ubiquitin proteasome system via the Keap1/Cullin complex. During oxidative stress, Keap1 modification prevents nrf2 degradation, which translocates into the nucleus (Robledinos‐Antón et al., 2019). In our study, isoprostanes concentration and nrf2 expression are not impacted by LPS under any experimental conditions. These results are surprising since, in human carcinoma cells, IL‐6 can be responsible for the activation of the nrf2 pathway (Matsuoka et al., 2016). This may differ in H9c2 ventricular cardiomyoblasts cells. However, in our study, we determined the expression of nrf2 and not its active fraction. Further studies are needed to determine the involvement of nrf2 in the antioxidant response of H9c2 cells following LPS exposure. Taken together, these results suggested that exposure to 5 μg/mL LPS for 24 h did not appear to induce major oxidative stress.

JNK and p38 belong to the MAPK (Mitogen‐activated protein kinase) family and are involved in a wide spectrum of biological responses. They are able to phosphorylate transcription factors in response to stimuli in order to enable gene expression. In physiological conditions, MAPK can improve cell survival, but when homeostasis is disturbed, they are activated in a prolonged way leading to chronic inflammation and apoptosis (Craige et al., 2019). Following the binding of LPS to TLR4, TAK1 is recruited and activates JNK and p38, leading to the expression of pro‐inflammatory cytokines (Sato et al., 2005). Our results showed that exposure to 5 μg/mL of LPS led to a trend toward increased JNK activation (*p* = 0.06) in the control group. Spi and PC showed a significant increase of JNK activation in the presence of LPS, while Se and SeSP remain stable. These results indicate that the increase in IL‐6 expression is not solely dependent on the JNK pathway. The effect of PC in reducing IL‐6 expression seems to act at another level than the activation of JNK. Moreover, Se seems to be able to inhibit JNK activation in the presence of LPS, contributing to a long‐lasting anti‐inflammatory effect. These results seem to be in agreement with those of Yang and coworkers who demonstrate that stimulation of the TLR4 receptor with 0.4 mM palmitic acid for 4 h on H9c2 cells leads to an increase in TLR4‐dependent JNK activation (Yang et al., 2021). In contrast, our results differ regarding p38. Indeed, no significant difference appeared in our model for all conditions except for phycocyanin. From 0 μg/mL of LPS, phycocyanin seemed to significantly increase p38 activation, while exposure to 5 μg/mL of LPS leads to a reduction. Interestingly, in an ischemia/reperfusion model, PC showed an ability to reduce p38 activation (Khan et al., 2006) while in a human breast cancer cell line (MDA‐MB‐231) PC is able to increase p38 activation leading to apoptosis (Jiang et al., 2018). The effect of PC on MAPK is still largely unknown, and further studies are needed.

**FIGURE 8:**
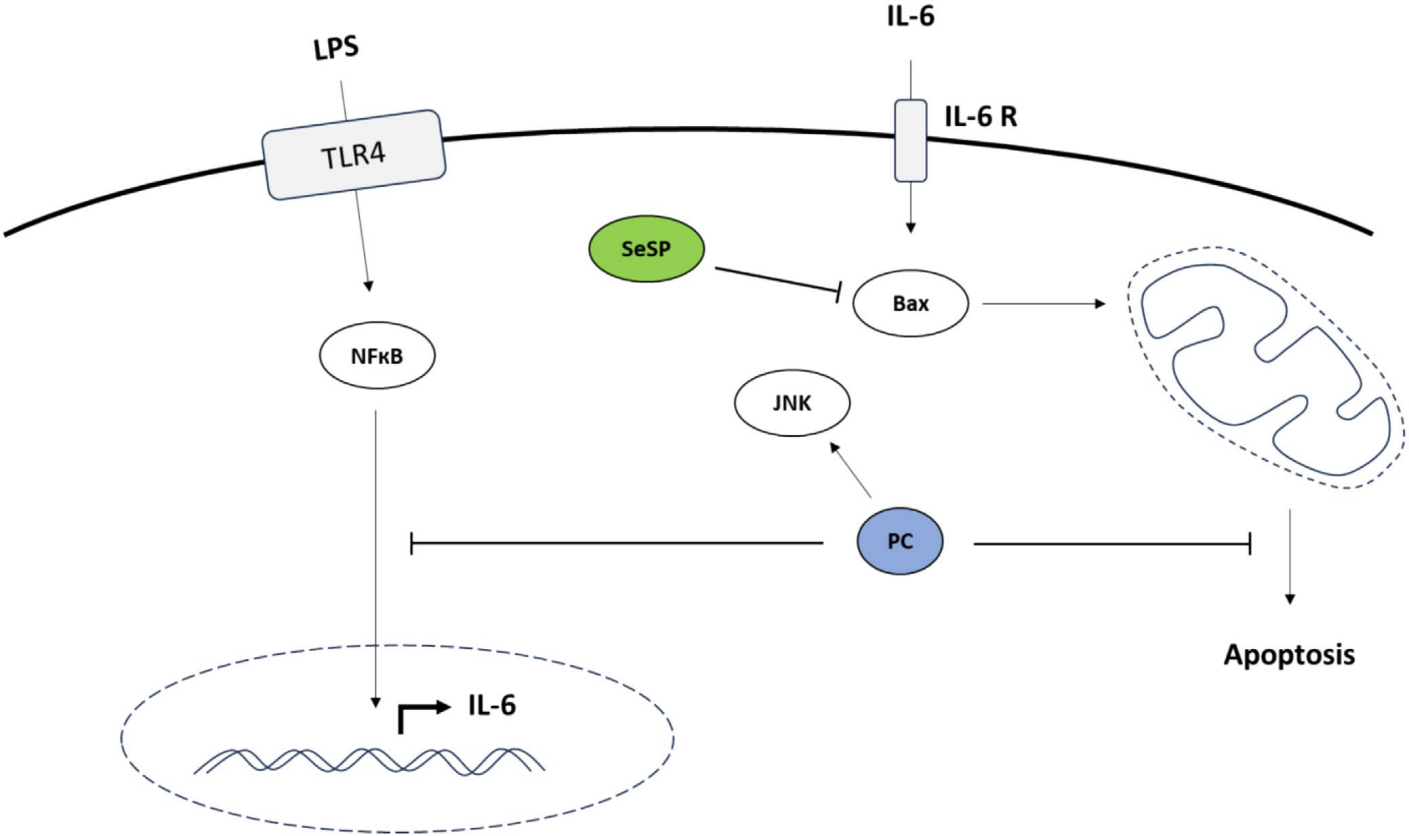
Overview of the cellular pathways impacted by LPS and the regulatory effects of PC and SeSP on these pathways in H9c2 cardiomyoblasts.

Principal component analysis (that allows to look at the parameters evolution without quantified statistics) revealed that in the absence of supplementation, exposure to 5 μg/mL LPS induced two distinct groups of parameters. Indeed, IL‐6 was closely correlated with Bax, GPx activity, Caspase 1, and p65 S536 phosphorylation. On the other hand, if inflammation seemed to be closely linked with the increase of pro‐apoptotic proteins, it seemed to be completely independent of oxidative stress parameters except for GPx and independent of caspase 3 activity. Thus, apoptosis could occur in a caspase 3‐independent signaling. However, the addition of Se at 0.5 μM led to a disruption of these parameters. Indeed, Bax and IL‐6 were still highly correlated, but GPx activity became independent of the generated inflammation. Since GPx is a selenoprotein, selenium administration could have influenced its activity. However, selenium did not influence nrf2, isoprostanes, bcl‐2, or caspase 3. Furthermore, selenium showed that phosphorylation of p65 S536 is negatively correlated with IL‐6 expression, as highlighted by the work of Ghosh and coworkers in 2010. In the presence of Spi at 2.5 μg/mL, the evolution of parameters was more scattered. Indeed, Spi seemed to not affect the correlation between Bax and IL‐6 and confirms once again the negative correlation between IL‐6 and p65 S536 phosphorylation. On the other hand, an action of spirulina on Caspase 1 and isoprostanes could be observed. Inflammation became caspase‐1 independent, suggesting a potential effect of spirulina on the release of pro‐inflammatory cytokines such as IL‐1β following LPS exposure. For its part, SeSP seemed to not impact the evolution of Caspase 3, Bcl‐2, nrf2, and isoprostanes. It also seemed to have no action on caspase 1. However, it retained the negative correlation between S536 phosphorylation and IL‐6 expression. Phycocyanin did not seem to affect the evolution of isoprostanes, bcl‐2, nrf2, and caspase 3 while inflammation became caspase 1 and p65 S536 phosphorylation independent. Thus, the observed effects of spirulina could be due to phycocyanin. A schematic summary of the key results is shown in Figure 8.

In conclusion, exposure of 5 μg/mL of LPS in H9c2 cells resulted in the release of IL‐6, which subsequently led to an increase in the pro‐apoptotic protein Bax. Moreover, the action of LPS did not seem to impact oxidative stress and some apoptotic parameters such as caspase 3 and Bcl‐2. On this basis, Selenium exerts an anti‐inflammatory effect by inhibiting the increase of JNK activation but does not impact apoptotic or oxidative stress parameters. It also acts on the phosphorylation of p65 S536. Spirulina has an effect on caspase 1 and p65 S536 phosphorylation. SeSP has a high amount of IL‐6 without LPS but reduced the increase of Bax after LPS exposure. It could limit apoptosis in H9c2 cells. In our experimental conditions, Phycocyanin appears to be the supplementation that brings the most benefits. Indeed, it reduces inflammation by reducing IL‐6, caspase 1, but also apoptosis by a non‐significant trend to reducing Bax.

## AUTHOR CONTRIBUTIONS

Castel T., Théron M., Pichavant‐Rafini K., Léon K. conceived and designed the experiments and contributed to the writing and revising of the article manuscript. Gandubert C. contributed to the acquisition, the analyses of the data. All authors have seen and approved the final manuscript.

## FUNDING INFORMATION

This research was not supported by any grant.

## CONFLICT OF INTEREST STATEMENT

The authors declare no conflicts of interest.

## ETHICS STATEMENT

Ethical approval was not required for this study.

## Data Availability

The authors declare that data and material are available upon reasonable request.
